# Unexpected Promotion of Bone Regeneration by Inhibition of BMPR1A‐Mediated BMP Signalling

**DOI:** 10.1111/cpr.70204

**Published:** 2026-04-06

**Authors:** Zihao Zhou, Yun Zhai, Xiaochen Liu, Jiaojiao Shao, Jiani Zhou, Zhaoyang Li, Jiale He, Shuxian Lin, Qi Zhang

**Affiliations:** ^1^ Shanghai Engineering Research Center of Tooth Restoration and Regeneration & Tongji Research Institute of Stomatology & Department of Endodontics, Shanghai Tongji Stomatological Hospital and Dental School Tongji University Shanghai China; ^2^ Shanghai Engineering Research Center of Tooth Restoration and Regeneration & Tongji Research Institute of Stomatology & Department of Prosthodontics, Shanghai Tongji Stomatological Hospital and Dental School Tongji University Shanghai China

**Keywords:** BMPR1A, bone regeneration, Osteoprogenitor cells, proliferation, targeted BMP regulation

## Abstract

Bone morphogenetic protein (BMP) signalling plays a pivotal role in bone regeneration by regulating osteoprogenitor cell (OPC) function, and BMPs have been widely used in clinical treatment. However, their limited specificity for OPCs often lead to side effects, highlighting that the regulatory mechanisms of BMP signalling remain to be further elucidated. BMPR1A, a key type I BMP receptor, has emerged as a critical regulator of bone development, yet its precise role in bone regeneration and downstream mechanisms remains unclear. Using OPC‐specific conditional knockout (cKO) and constitutively activated (CA) BMPR1A mouse, we found conditional knockout of BMPR1A in OPCs during the first 2 weeks of healing significantly accelerated bone regeneration. At the cellular level, BMPR1A knockout promoted the proliferation of OPCs, thereby accelerating bone regeneration in cKO mice. Mechanistically, BMPR1A knockout reduced ID1 expression, releasing its inhibition of TCF3, which in turn induced GNG4 expression and ultimately activated the PI3K–AKT pathway. Finally, a double‐knockdown cell line further demonstrated the role of the BMPR1A–ID1–TCF3–GNG4 signalling axis. This study reveals the function and mechanism of BMPR1A in bone regeneration and provides new insights for more precise BMP‐targeted strategies.

## Graphical abstract

BMPR1A downregulation reduces ID1, releases TCF3 to activate GNG4‐PI3K‐AKT signalling, thereby promoting OPC proliferation and bone regeneration.

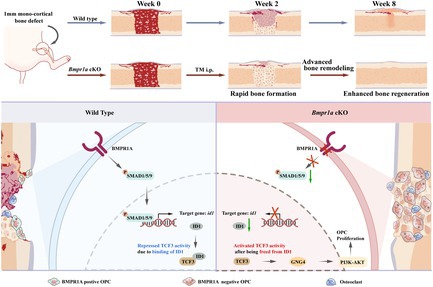

## 1 Introduction

Bone defects are common clinical injuries that are often accompanied by local or systemic dysfunction [1, 2]. Optimal therapy relies on enhancing the intrinsic regenerative capacity [1]. Bone regeneration is orchestrated by multiple signalling pathways and diverse cell types [3, 4]. Among these, the bone morphogenetic protein (BMP) pathway serves as a key driver of osteogenesis, primarily through its regulation of osteoprogenitor cells (OPCs) [5]. OPCs, a specialized subset of mesenchymal stem cells, act as the main effector cells in bone repair. They are self‐renewing and differentiating into osteoblasts, effectively guiding and initiating new bone formation [3, 4, 6, 7, 8, 9]. Conditional deletion of BMP2 in OPCs impairs fracture healing in mice [10], underscoring the indispensable role of BMP signalling in modulating OPCs‐mediated bone repair. Currently, BMP2 is the only approved osteoinductive ligand for bone repair, and it exhibits strong bone‐forming activity; however, its clinical application is limited by significant side effects, such as heterotopic ossification, acute inflammation, and osteolysis. These side effects mainly arise from its broad stimulation of multiple cell populations, including immune cells, myocytes, and stromal cells, while lacking the specificity to selectively target OPC [5]. Therefore, selectively targeting BMP signalling in osteoprogenitor cells may represent a promising approach to improve BMP‐based therapies while minimizing side effects.

The BMP signalling pathway involves three components: ligands (BMPs), receptors (bone morphogenetic protein receptors, BMPRs), and downstream SMAD effectors [11, 12, 13]. Transduction critically depends on the binding of BMP ligands to BMPRs, which initiate intracellular signalling cascades via SMAD proteins. Historically, research on bone defect repair has focused predominantly on BMP ligands, including BMP2, BMP4, and BMP7 (Ref [5, 12, 14]). However, the broad distribution of BMPR subtypes across diverse cell types limits the specificity of BMP ligands, which can bind multiple receptors, activate divergent pathways, and trigger unintended side effects [5]. Owing to these limitations, the spatiotemporal heterogeneity of BMPR subtypes presents a promising therapeutic opportunity. As the key mediators of BMP signal transduction, BMPR subtypes exhibit remarkable variability in their spatial distribution, temporal expression patterns, and abundance across different cells and tissues [7, 14]. These findings suggest that identifying BMPR subtypes that are specifically enriched in OPCs and functionally critical for bone regeneration may provide a molecular target for achieving cell‐specific modulation of BMP signalling.

BMPR functions as a heteromeric complex comprising type I (BMPRI) and type II (BMPRII) receptors. Within this complex, BMPRI acts as the primary signalling mediator since the intracellular signalling cascade is initially triggered by the phosphorylation of its intracellular glycine/serine (Gly/Ser)‐rich domain [7, 11, 14]. Among all BMPRI subtypes, ACVR1 and BMPR1A are the major subtypes that are expressed on the membranes of OPCs [14]. Notably, ACVR1 is also highly expressed on muscle cells, where constitutive activation causes fibrodysplasia ossificans progressiva (FOP), a genetic disease characterized by progressive heterotopic ossification [14, 15]. The pathological association between ACVR1 and ectopic bone formation represents a significant risk in BMP2‐induced bone regeneration therapies. In contrast, accumulating evidence suggests that BMPR1A is a key target for precisely modulating OPC function to enhance bone regeneration. As an osteoprogenitor surface marker by Takamitsu et al. [16], BMPR1A was demonstrated to have structural specificity through features such as Phe85 in its extracellular domain, enabling its high‐affinity binding to BMP2 [17, 18]. In addition, studies focused on bone development revealed a critical role of BMPR1A in regulating osteogenesis, suggesting the potential functions of BMPR1A in bone regeneration, including the following: (1) Proliferation control: the suppression of BMPR1A significantly impairs the proliferative capacity of osteolineage cells, including osteoprogenitors, osteoblasts, and osteocytes. (2) Differentiation modulation: Stage‐dependent effects on osteogenic differentiation have been reported in genetic studies. Guo et al. demonstrated that BMPR1A knockout driven by *Gli1‐Cre* upregulated *Alp*, *Sp7*, and *Dmp1* expression [19], whereas BMPR1A deletion driven by 3.2 kb *Col1‐Cre* resulted in impaired osteogenic differentiation and reduced mineralization [20]. Given its functions and the distinct microenvironments between osteogenesis and bone regeneration, elucidating the biological role and mechanism of BMPR1A during bone regeneration is essential. However, its biological roles and underlying mechanisms remain largely unclear.

In this study, we used transgenic mice to investigate the role and mechanisms of BMPR1A in OPCs during bone regeneration. Conditional knockout of BMPR1A in OPCs during the first 2 weeks of repair enhanced bone formation and remodelling. In vitro studies demonstrated that BMPR1A strongly regulated OPC proliferation. Mechanistically, BMPR1A activation triggers the nuclear translocation of SMAD1/5/8, which induces the expression of inhibitor of DNA‐binding 1 (ID1). ID1 subsequently binds to transcription factor 3 (TCF3), inhibiting its biological activity. However, *Bmpr1a* cKO increased the expression level of ID1, thereby releasing TCF3 and activating the GNG4‐PI3K–AKT signalling axis, which drives OPC proliferation and bone regeneration. These findings uncover a previously unrecognized function of BMPR1A in bone regeneration, offering insight into BMP‐based strategies to improve bone repair.

## 2 Methods

### 2.1 Animals

All the mice were maintained on a C57BL/6J background in a specific pathogen‐free (SPF) facility under a 12/12 h day/night illumination cycle. 3.6 kb *Col1a1*‐Cre^ERT2^ mice, R26R^Td‐tomato^ mice, *Bmpr1a* ^*f/f*^ mice and pmes‐ca*Bmpr1a* mice were used. All animals were bred according to the National Institutes of Health's Guide for Care and Use of Laboratory Animals. All the animal studies were carried out in accordance with the guidelines of the Institutional Animal Care and Use Committees of Tongji University ([2022]‐DW‐11) and followed all the ARRIVE recommendation (Animal Studies: Reporting of In Vivo Experiments) guidelines. The following mouse strains were used: 3.6 kb *Col1a1*‐Cre^ERT2^ mice: Kindly provided by Professor J.Q. Feng (Texas A&M University, USA) and Professor Dr. Lin Chen (Third Military Medical University, Chongqing, China), this strain expresses tamoxifen‐inducible Cre recombinase under the control of the 3.6 kb *Col1a1* promote [21, 22]. *Bmpr1a* ^*f/f*^ mice: Kindly provided by Professor Yuji Mishina (University of Michigan, USA), this strain harbours loxP sites flanking exon 2 of the *Bmpr1a* gene [23]. pmes‐ca*Bmpr1a* mice: Kindly provided by Professor Yan‐ding Zhang (Fujian Normal University, China), this strain carries a loxP‐stop‐loxP‐regulated constitutively active BMPR1A mutant [24, 25]. R26R^Td‐tomato^ reporter mice: The Jackson Laboratory (Stock No: 007914). For breeding, 3.6 kb *Col1a1*‐Cre^ERT2^ mice were mated with R26R^Td‐tomato^, *Bmpr1a* ^*f/f*^, or pmes‐ca*Bmpr1a* mice to generate lineage tracing mice, osteoprogenitor‐specific BMPR1A conditional knockout (cKO), and conditional activation (BMPR1A CA) mice, respectively. Male mice were used for all experiments.

### 2.2 Femur Defect Model

A single‐layer femoral cortical bone defect model was constructed following the methods of Zhang et al. [26]. Initially, the mice were anaesthetized via an intraperitoneal injection of sodium pentobarbital and secured to the surgical platform. The hair from the operative area was subsequently removed, and the skin of the femur was disinfected. Next, the skin was incised, the muscle was bluntly dissected, and the periosteum was gently lifted to expose the surface of the femur. Subsequently, we used a dental drill to create a single‐layer cortical bone defect with a diameter of 1 mm in the centre of the femur. Afterward, we cleaned and sutured the wound and administered penicillin by intraperitoneal injection. Moreover, we injected tamoxifen every 3 days until the time of harvest. For experiments at various stages of bone regeneration, we had three mice per time point, whereas for the experiment at 2 weeks post‐defect (PD), the number of mice per group was increased to six.

### 2.3 Micro‐CT Analysis

Dissected femurs were fixed in 4% paraformaldehyde (PFA) for 48 h. The femurs were scanned via a micro‐CT 50 (Scanco Medical, Zurich, Switzerland) at a resolution of a 14 μm slice increment with a voltage of 70 kV and a current of 200 μA. Sixty slices of the bone defect area were reconstructed for statistical analysis. The parameters of trabecular bone volume over total volume (BV/TV), trabecular bone number (Tb.N), trabecular bone thickness (Tb.Th), and trabecular bone separation (Tb.Sp) were quantified according to standard procedures.

### 2.4 Histological Assessment

Femurs were decalcified in 10% ethylenediaminetetraacetic acid (EDTA) (pH 7.4) at 4°C for 4 weeks. After dehydration through a series of graded ethanol solutions, the samples were embedded in paraffin and cut into 4‐μm‐thick sections. The sections were then deparaffinized in xylene and rehydrated in a descending series of ethanol concentrations. Haematoxylin and eosin (H&E) (Sangon Biotech, Shanghai, China) staining was performed for morphological evaluation. The sections were stained with Masson's Trichrome, Von Kossa, and Trap to assess changes in the three groups.

### 2.5 Immunofluorescence Staining and Immunohistochemistry

Immunofluorescence staining was performed in mice and cell lines. Paraffin and frozen mouse sections were used. The preparation method for the paraffin sections was previously described. For frozen sections, femurs were embedded in optimal cutting temperature (OCT) compound (Sakura, Taizhou, China) and sectioned at a thickness of 10 μm after decalcification. Cultured cells were fixed in 4% PFA for 15 min and subsequently washed twice with phosphate‐buffered saline (PBS). Antigen retrieval was performed via the use of hyaluronidase or 0.1% trypsin in mice. All the samples were blocked in 5% fetal bovine serum (FBS) in PBS‐T (0.1% Triton X‐100 in PBS) or goat serum at room temperature and then incubated overnight at 4°C with primary antibodies. The membrane was then incubated with a secondary fluorochrome‐conjugated antibody for 60 min. For cells, the actin cytoskeleton was stained with AlexaFluor 488 phalloidin (Yeasen, China, 1:1000) or rhodamine‐conjugated phalloidin (Yeasen, China, 1:100). All the nuclei were counterstained with DAPI for 5 min. Finally, the sections were mounted with anti‐fade prolong reagent (Invitrogen), and images were obtained with an Eclipse Ni‐U microscope (Nikon, Japan) or Nikon TI2‐E + A1 R confocal microscope (Nikon, Japan).

Paraffin sections were used for immunohistochemical staining. Hyaluronidase was utilized for antigen retrieval. A 3% hydrogen peroxide (H_2_O_2_) solution was used to reduce endogenous peroxidase activity. Then, the sections were blocked as described previously. The sections were subsequently incubated with primary antibody at 4°C overnight. Biotinylated secondary antibodies were applied, and the samples were further processed via streptavidin peroxidase and a diaminobenzidine (DAB) detection kit (MXB, China) according to the instructions. The primary antibodies used were rabbit anti‐BMPR1A (Abcam, ab264043), rabbit anti‐PDGFR a (R&D systems, AF1062), rabbit anti‐SP7 (Abcam, EPR21034), rabbit anti‐OCN (Affinity, DF12303), rabbit anti‐OPN (Affinity, AF0227), anti‐DMP1, mouse anti‐PCNA (Abcam, ab29), rabbit anti‐BAX (Boster, A00183), rabbit anti‐BAK (Boster, PB0506), mouse anti‐ID1 (Proteintech, 67,827–1‐Ig), rabbit anti‐TCF3 (Proteintech, 67,140–1‐Ig), and rabbit anti‐GNG4 (Proteintech, 13,780–1‐AP). The secondary antibodies used were anti‐mouse IgG (A‐10684, Invitrogen) and anti‐rabbit IgG (A‐11072, 1:1000; Invitrogen).

### 2.6 Cell Culture and Transfection

The C3H10T1/2 clone 8 MSC line was cultured as previously described. The cells were maintained at 37°C and 5% CO_2_. Dulbecco's modified Eagle's medium (DMEM) (Gibco, Shanghai, China) supplemented with 5% FBS was used.

siRNAs and plasmids were transfected via Lipofectamine 3000 (Invitrogen, Waltham, MA). siRNAs targeting *Bmpr1a, Id1, Id2, Tcf3*, and *Gng4* were purchased from Tsingk (Shanghai, China), and overexpression plasmids were purchased from Genechem Co. Ltd. (Shanghai, China). The sequences of the siRNAs are shown in Table S2. Transfection procedures were performed in accordance with the Lipofectamine 3000 reagent instructions. Total RNA was extracted at 48 h after transfection, and protein was extracted at 72 h after transfection.

### 2.7 Cell Proliferation Assay

Cell proliferation was assayed via a cell counting kit‐8 (CCK8), 5‐ethynyl‐2′‐deoxyuridine (EdU; Beyotime, Shanghai, China), and colony formation assays.

For the CCK8 assay, cells were placed into 96‐well plates at 3000 cells per well and subsequently incubated under identical conditions. CCK8 solution was added to the wells, which were then incubated for 1 h. The absorbance at 450 nm was determined via a microplate reader (Bio‐Tek, Hercules, CA, USA).

Cells or paraffin sections were used for the EdU assay. EdU was dissolved in PBS and administered 2 h before harvest (500 μg per mouse and 20 nM for cells). BeyoClick EdU‐594 (Beyotime, Shanghai, China) was used to detect EdU. All procedures were performed according to the instructions of the EdU kit (Beyotime, Shanghai, China).

For the colony formation assay, cell lines were transfected with siRNA, and single‐cell suspensions were subsequently prepared with 0.25% trypsin and seeded in 6‐well plates at 500 cells per well. The cells were cultured for 14 days. All the samples were subsequently fixed with 4% PFA for 15 min and stained with 0.5% crystal violet for 10 min. The 6‐well plates were photographed under a light microscope.

### 2.8 Cell TUNEL Assay

A TUNEL kit was used to evaluate apoptosis in calluses. Paraffin‐embedded sections were treated as described previously. All the sections were subsequently incubated with the TUNEL reaction mixture for 1 h at 37°C, after which the nuclei were counterstained with DAPI. Finally, the sections were mounted with an anti‐fade agent and examined via fluorescence microscopy.

### 2.9 RNA Extraction and Quantitative Real‐Time PCR

Total RNA was extracted via TRIzol (Takara, China) via cDNA Synthesis SuperMix for quantitative polymerase chain reaction (qPCR) with gDNA Eraser (Yeasen, China). To quantify the mRNA levels, real‐time qPCR was performed via qPCR SYBR Green Master Mix (Yeasen, China) on a LightCycler96 (Roche, Switzerland) device. The data were normalized to the housekeeping gene *β‐actin*, and relative expression was evaluated via the 2^−ΔΔCt^ method. The primers were designed via Primer 6 software, and the primer sequences are listed in Table S1.

### 2.10 Protein Extraction and Western Blotting

The calluses or cells were lysed with ice‐cold RIPA buffer and then sonicated on ice. After a 30‐min incubation period on ice, the lysate was centrifuged at 12,000 × g for 30 min at 4°C. The supernatants were collected, and the protein content was assessed via a BCA protein assay kit. Equal amounts of protein samples were separated on 10% SDS–polyacrylamide gels (SDS–PAGE) and transferred to polyvinylidene fluoride (PVDF) membranes (Millipore, USA). The membranes were then probed with primary antibodies against SMAD1/5/9 (Affinity, AF0614, 1:2000), phospho‐SMAD1/5/9 (Affinity, AF8313, 1:2000), PI3K (Affinity, AF6241,1:1000), phospho‐PI3K (Affinity, AF3241, 1:2000), AKT (CST, 9272, 1:2000), phospho‐AKT (CST, 4060, 1:2000), ID1 (Proteintech, 67,827–1‐Ig, 1:2000), TCF3 (Proteintech, 67,140–1‐Ig, 1:2000) and beta‐Actin (Affinity, T0022, 1:10000). The membranes were washed three times with Tris‐buffered saline with Tween‐20 (TBST) buffer for 10 min each, followed by a 2‐h incubation with a secondary antibody conjugated to horseradish peroxidase (HRP) (Beyotime, Shanghai, China). Enhanced chemiluminescence (ECL, Beyotime, Shanghai, China) and detection equipment were used to identify protein bands. The intensity of each protein band was subsequently measured via image analysis techniques (ImageJ).

### 2.11 RNA‐Seq

Total RNA was extracted from samples via the Qiagen RNeasy Mini Kit, and its quality was assessed via a NanoDrop spectrophotometer and Agilent 2100 Bioanalyzer. mRNA was enriched via oligo‐dT magnetic beads, followed by fragmentation and cDNA synthesis. Double‐stranded cDNA was end‐repaired, A‐tailed, and adapter‐ligated with unique molecular indices. PCR enrichment was performed, and the libraries were purified and validated via an Agilent 2100 Bioanalyzer. Sequencing was conducted on an Illumina NovaSeq platform to generate paired‐end reads. After sequencing, the data were processed for quality control, alignment, and gene expression quantification, with differential expression analysis performed via the DESeq2 R package (1.16.1)/edge R package (3.18.1). The *p* values were adjusted via the Benjamini & Hochberg–Bonferroni method. A corrected *p* value of 0.05 and an absolute fold change of 2 were set as the thresholds for significantly differential expression. Gene Ontology (GO) and Kyoto Encyclopedia of Genes and Genomes (KEGG) enrichment analyses of the DEGs were performed via the clusterProfiler R package. PPI analysis of the differentially expressed genes was performed via the STRING database, which includes known and predicted protein–protein interactions.

### 2.12 Flow Cytometry

Femurs were dissociated by mechanical and enzymatic dissociation according to a published protocol [27]. Femurs were briefly digested with 0.2% collagenase type I at 37°C for 30 min. Flow cytometry was performed using a BD FACSAria III (Becton Dickinson). The total dissociated cells were blocked with rat immunoglobulin G and stained with fluorochrome‐conjugated antibodies against CD45 (BioLegend, 103,154, 1:400), CD31 (BioLegend, 102,528, 1:400), Ter119 (BioLegend, 116,223, 1:200), and PDGFRα(R&D systems, AF1062, 1:400) for fractionation by FACS.

### 2.13 Luciferase Reporter Assay

To verify the transcriptional regulation of *Gng4* by TCF3, the core region of the mouse *Gng4* gene promoter containing the predicted TCF3‐binding site was cloned and inserted into the pGL3‐Basic vector to generate the Gng4‐promoter‐Luc reporter plasmid. C3H10T1/2 cells were seeded in 24‐well plates and co‐transfected at 70%–80% confluence using Lipofectamine 3000. The transfection mixture per well contained *Gng4*‐promoter‐Luc reporter plasmid, TCF3 overexpression plasmid or empty vector control, and the Renilla luciferase internal control plasmid pRL‐TK. After 48 h of transfection, firefly and Renilla luciferase activities were measured sequentially using the Firefly Luciferase Reporter Gene Assay Kit II (Beyotime. RG007S). Relative luciferase activity was calculated by normalizing firefly luciferase activity to Renilla luciferase activity.

### 2.14 Statistical Analysis

The statistical data are presented as the means ± SD. Comparisons between the two groups were analysed via a two‐tailed, unpaired Student's *t*‐test. One‐way ANOVA was used when the data were compared among multiple groups. GraphPad PRISM v.9.0.1 was used for statistical analysis. *p* < 0.05 was considered statistically significant.

## 3 Results

### 3.1 BMPR1A ^+^ Osteoprogenitors Are Transiently Enriched in the Callus

**FIGURE 1:**
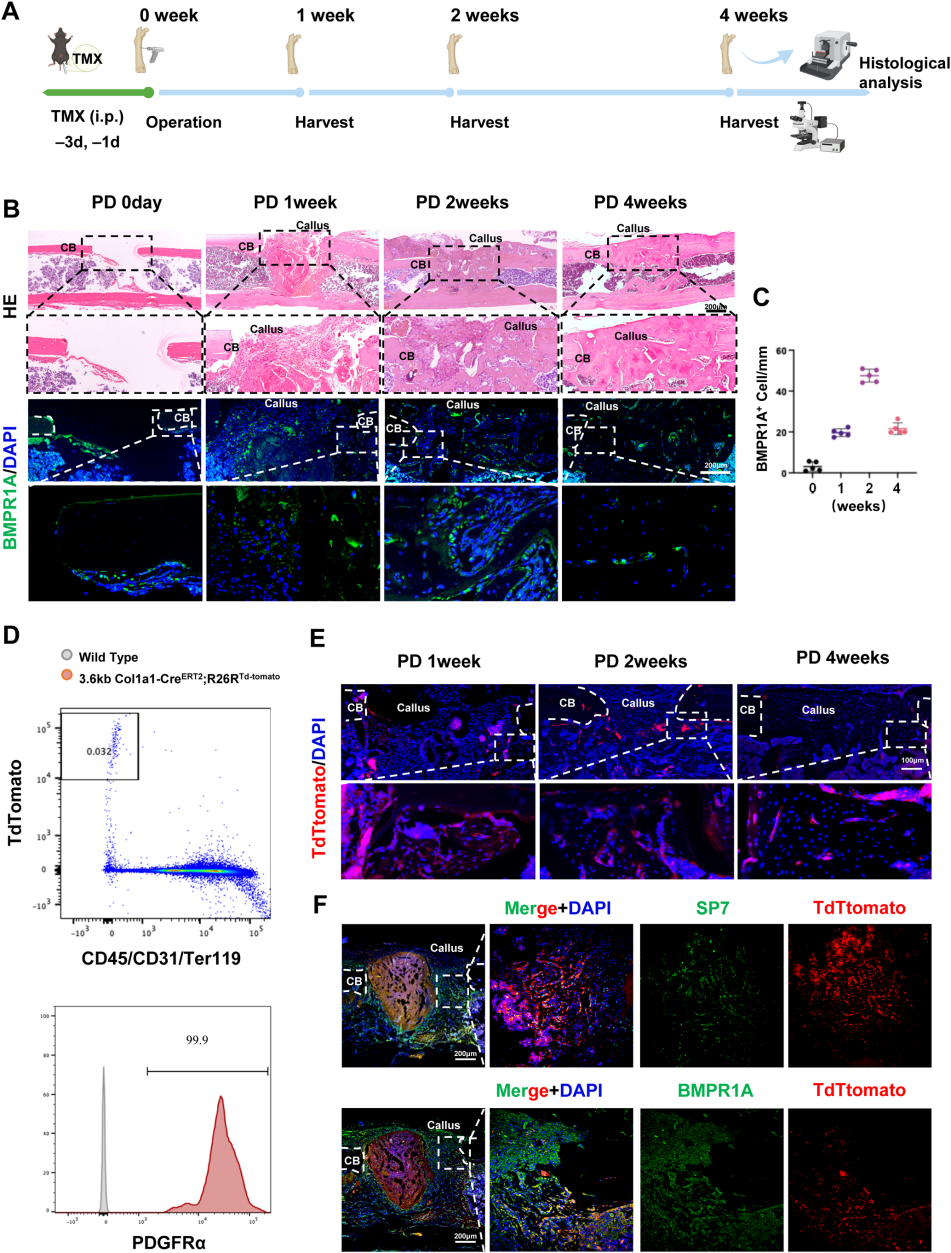
Expression of 3.6 kb *Col1a1* ^+^ *BMPR1A* ^+^ OPCs during bone regeneration. (A) Timeline of the operation, TM injection and harvest. (B) HE and Immunofluorescence images of BMPR1A at 0 days, 7 days, 14 days, and 28 days after the operation in the control group. CB, Cortical Bone. (C) Quantification of BMPR1A‐positive cells in the callus (*n* = 5). (D) Flow cytometry analysis results showing the percentage of 3.6 kb *Col1*‐positive cells and colabelling with the bone progenitor cell marker PDGFR (*n* = 3). (E) Immunofluorescence images of tracing mice in which 3.6 kb col1‐positive cells were detected during bone repair after the operation. CB, Cortical Bone; NB, new bone. (F) confocal Immunofluorescence images of SP7 and BMPR1A in callus in tracing mice at 7 days post‐operation.

Bone regeneration can be divided into three stages: fibrous tissue formation (postoperative day, PD 0–1 week), callus formation (PD 2 weeks), and remodelling (PD 4 weeks), which guided our study (Figure 1A). To investigate the role of BMPR1A in bone regeneration, 1.0‐mm monocortical femoral defects were generated in 12‐week‐old male mice, and samples were collected at PD 0 day, 1 week, 2 weeks, and 4 weeks to cover the full stage of regeneration. BMPR1A was specifically expressed on the membrane of cells surrounding newly formed bone, and its expression dynamically increased during bone formation, reaching a peak at 2 weeks post‐injury (Figure 1B,C), indicating its localization on OPCs or preosteoblasts. To selectively manipulate BMPR1A activity in this population, we reviewed previous studies and selected 3.6 kb *Col1a1*‐Cre^ERT2^, which has been reported for OPCs labelling [28], and we generated 3.6 kb *Col1a1*‐Cre^ERT2^; R26R^Td‐tomato^ reporter mice. Flow cytometry analysis revealed that 3.6 kb *Col1a1* ^+^ cells, which accounted for ~0.4% of bone marrow mesenchymal stem cells, coexpressed PDGFRα, a canonical osteoprogenitor marker on the membrane (Figure 1D and Figure S1A). Immunofluorescence staining further confirmed that 3.6 kb *Col1a1* ^+^ cells highly overlapped with PDGFRα^+^ cells, providing a precise cellular basis for all subsequent observations of OPC function (Figure S1B). Tamoxifen was injected 3 days and 1 day before the operation, and the mice were sacrificed at 1 week, 2 weeks, and 4 weeks post‐operation. Frozen sections of the injury site revealed a gradual increase in 3.6 kb *Col1a1* ^+^ cells over time, which were predominantly located around newly formed bone (Figure 1E) and spatially overlapped with *Bmpr1a* ^+^ cells. SP7 immunofluorescence staining further confirmed the osteogenic potential of 3.6 kb *Col1a1* ^+^ cells (Figure 1F). Immunofluorescence staining also revealed colocalization of BMPR1A and 3.6 kb *Col1a1* ^+^ Cre, indicating a correlation between BMPR1A and OPCs (Figure 1F).

These findings confirmed that 3.6 kb *Col1a1* ^+^ cells represent a subset of osteoprogenitors characterized by high expression of BMPR1A on their cell membrane. These cells exhibit osteogenic differentiation potential and are actively involved in bone regeneration.

### 3.2 BMPR1A Downregulation in 3.6 Kb *Col1a1* ^+^ OPCs Accelerates the Process of Bone Regeneration

To investigate BMPR1A in bone regeneration, we conditionally knocked out or activated BMPR1A in 3.6 kb *Col1a1* ^+^ OPCs by crossing 3.6 kb *Col1a1*‐Cre^ERT2^ mice with *Bmpr1a* ^*f/f*^ or pmes‐ca‐*Bmpr1a*, generating OPC‐specific *Bmpr1a* conditional knockout (cKO) and constitutively activated (CA) mice (Figure S1C). Tamoxifen was injected during different post‐injury intervals (0–1, 0–2, 0–3, or 0–4 weeks), and femurs were harvested at 4 weeks. Micro CT, X‐ray and H&E staining revealed that BMPR1A inhibition during the early 0–2‐week period markedly enhanced bone repair compared with later treatment groups, characterized by continuous cortical bridging and lower callus volume on micro‐CT (Figure S2A–C), suggesting that 0–2 weeks is critical for BMPR1A‐mediated regeneration. To further characterize this critical window, we examined the molecular dynamics of normal bone regeneration at postoperative weeks 1, 2, and 4 in control mice. At week 1 post‐injury, Sp7^+^ cells appeared abundantly at the defect edges (Figure S3A), indicating active initiation of osteoblast lineage recruitment. While by week 4, OCN showed strong positivity in the newly formed bone matrix (Figure S3A), signifying entry into the mineralization phase.

**FIGURE 2:**
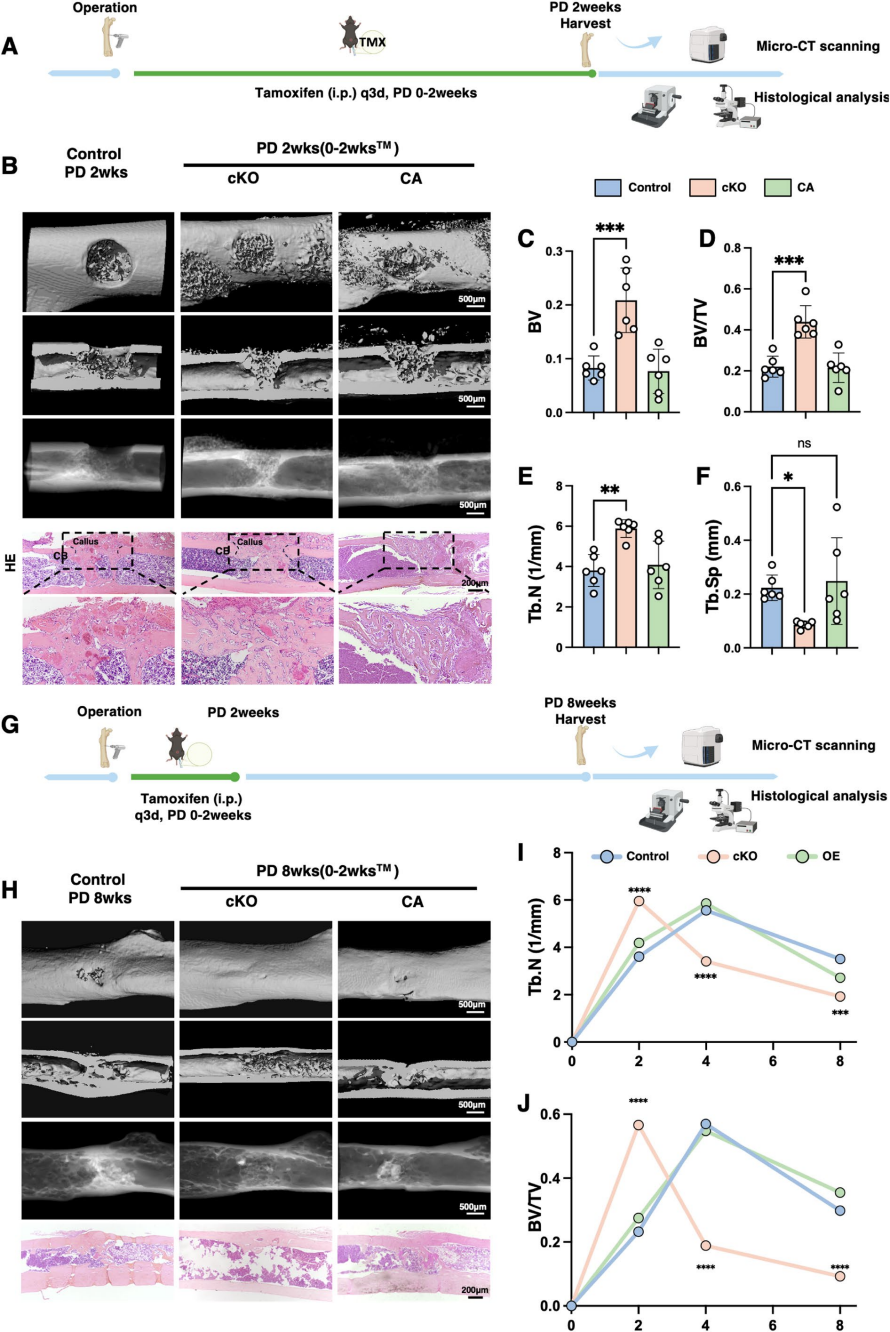
BMPR1A downregulation in 3.6 *kb Col1a1* ^+^ OPCs accelerated the process of bone regeneration. (A) Timeline of the operation and TM injection. (B) Micro‐CT, X‐ray and HE images of the 3 groups at 2 weeks after the operation. CB, Cortical Bone. (C–F) Statistical analysis of BV, BV/TV, Tb.N and Tb.sp. from micro‐CT scans of the calluses of the three groups at 2 weeks after the operation. *p < 0.05, **p < 0.01, ***p < 0.001 versus control, *n* = 6. (G) Timeline of the operation and TM injection. (H) Micro‐CT, X‐ray and HE images of cKO and CA 0–2 weeks femurs harvested at 8 weeks after the operation compared with the control group. CB. Cortical Bone. (I, J) Line chart of Tb.N and BV/TV during bone regeneration in the three groups in which BMPR1A expression was regulated at 0–2 weeks. ****p < 0.0001 versus control, *n* = 3.

Based on this critical window, we next examined the phenotype of BMPR1A inhibition at 2 weeks post‐operation. Contrary to expectations, BMPR1A inhibition during the early phase enhanced healing, implying that downregulation of BMPR1A‐mediated BMP signalling positively contributes to early repair. To confirm the hypothesis, fumers were harvested at 2 weeks for early‐stage analysis (Figure 2A). Rapid bone formation was detected in cKO mice. CT and X‐ray analyses revealed apparently increased new bone formation in cKO mice (Figure 2B). H&E staining revealed that the control group contained abundant osteoid representing incompletely mineralized new bone, whereas these were markedly reduced in the cKO mice (Figure 2B). More importantly, a bone bridge had formed well in cKO mice (Figure 2B, blue arrowhead). Quantitative analysis revealed increases in bone volume (BV, Figure 2C), bone volume/tissue volume (BV/TV, Figure 2D), and trabecular number (Tb.N, Figure 2E), coupled with a decrease in trabecular separation (Tb.Sp, Figure 2F), with no change in bone mineral density (BMD) and trabecular thickness (Tb.Th) (Figure S2D).

At 8 weeks, the calluses in the cKO group were nearly undetectable (Figure 2G,H), and the peak of Tb.N (Figure 2I) and BV/TV (Figure 2J) occurred at 4 weeks. These results demonstrated that BMPR1A inhibition during early repair (0–2 weeks) strongly enhances bone healing.

### 3.3 BMPR1A Downregulation in 3.6 Kb *Col1a1* ^*+*^ Cells Efficiently Promotes Bone Formation and Promotes Bone Remodelling During Osteoregeneration

**FIGURE 3:**
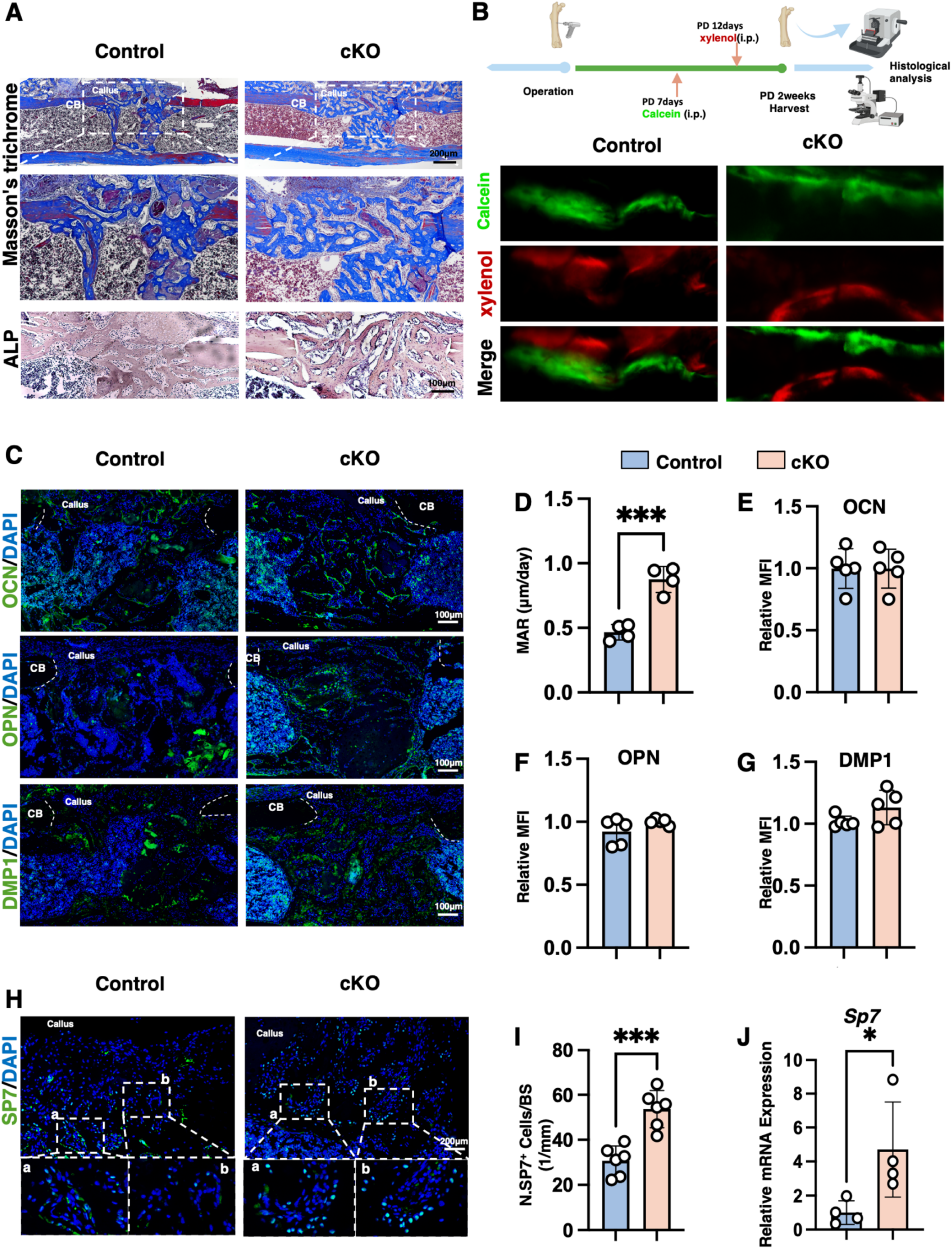
BMPR1A modulates the rate of bone formation during the first 2 weeks of the bone repair process. (A) Masson's trichrome in calluses at 2 weeks after the operation. CB, cortical bone. (B) Double‐labelling in calluses at 2 weeks after the operation. (C) Immunofluorescence images of OCN, OPN and DMP1 in calluses at 2 weeks after the operation. CB, cortical bone. (D) Statistical analysis of MAR in the injury area at 2 weeks after operation. ***p < 0.001, *n* = 4. versus relative control, *n* = 5. (E) RFI of OCN between cKO mice and control mice, *n* = 5. (F) RFI of OPN between cKO mice and control mice, *n* = 5. (G) RFI of DMP1 between cKO mice and control mice. (H) Immunofluorescence images of SP7 in calluses at 2 weeks after the operation. (I) Quantification of the number of SP7^+^ cells surrounding newly formed bone trabeculae in the two groups at 2 weeks after the operation. ****p < 0.0001 versus the control, *n* = 6. (J) mRNA levels of *Sp7* in the cKO and control groups at PD 2 weeks. *p < 0.05 versus the control, *n* = 4.

Given these unexpected results, we next explored the mechanisms be suppression of BMPR1A‐mediated BMP signalling enhances bone regeneration. Considering the dual regulatory role of BMPR1A, we first evaluated its influence on osteogenesis [3, 29]. Masson's trichrome showed enhanced bone formation in the callus of BMPR1A cKO mice compared with controls. (Figure 3A). Double labelling demonstrated a 1.88‐fold higher mineral apposition rate (MAR) in cKO mice (Figure 3B,D). To evaluate osteoclast function, Tartrate‐resistant acid phosphatase (TRAP) staining revealed increased TRAP‐positive osteoclasts within/near cKO calluses (Figure S3B), and immunofluorescence showed increased RANKL, whereas OPG remained unchanged (Figure S3B).

Based on the overall trends observed in cKO mice, we proposed that enhanced osteogenesis predominantly derived the accelerated regeneration. Furthermore, the expression of osteogenic markers (OCN and OPN, Figure 3C,E,F) remained unchanged or modestly upregulated (e.g., DMP1, Figure 3C,G). However, SP7 expression was strikingly elevated in cKO calluses (Figure 3H–J). The increase in the number of SP7^+^ cells differentiated from OPCs likely underpins the enhanced osteogenesis and rapid regeneration, warranting further studies.

### 3.4 BMPR1A Inhibition Enhances OPC Proliferation at the Early Stage of Osteoregeneration

**FIGURE 4:**
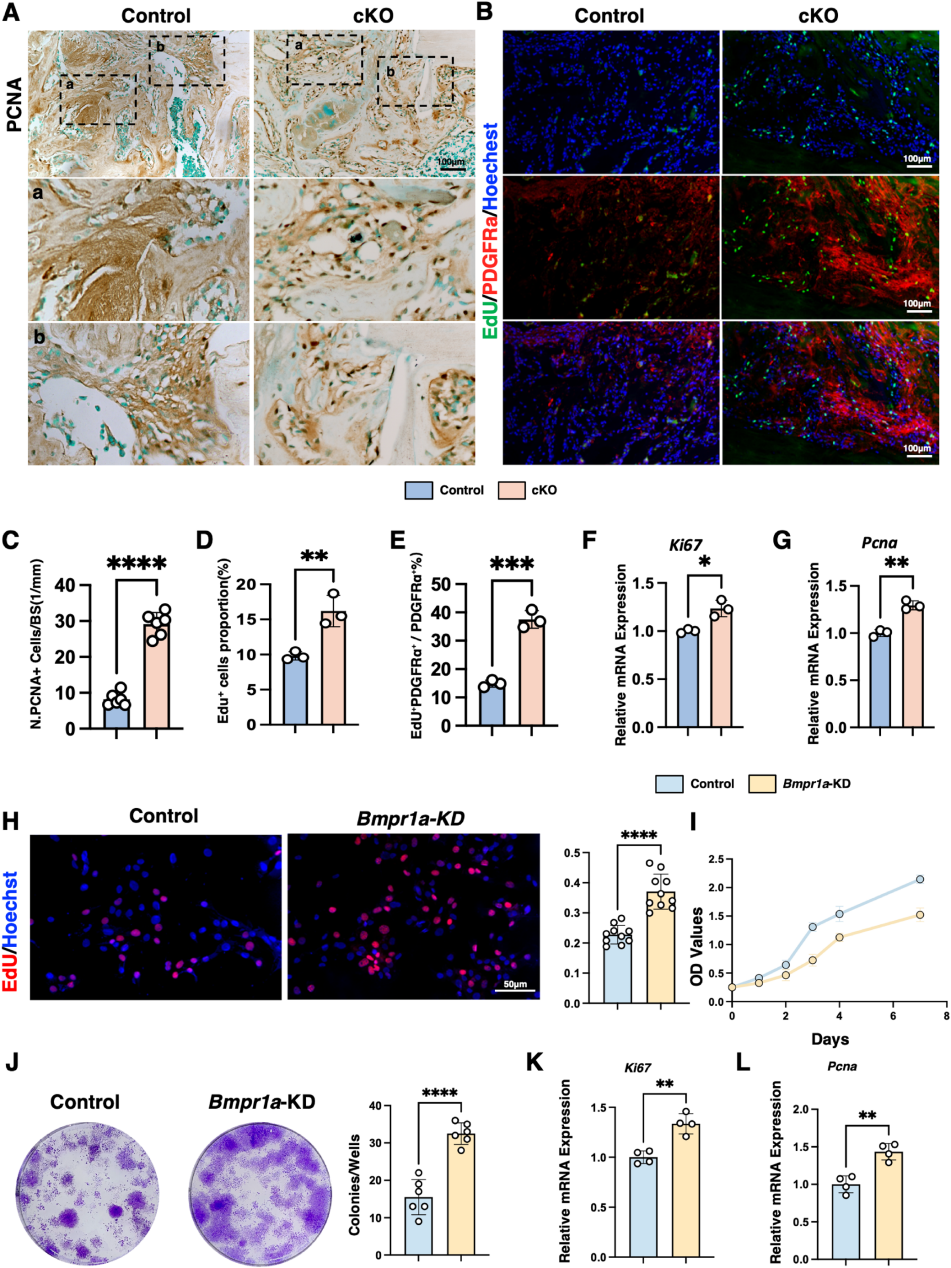
BMPR1A inhibition enhances OPC proliferation at the early stage of osteoregeneration. (A) Immunohistochemical Staining of PCNA in calluses at 2 weeks after the operation. (B) Immunofluorescence staining of EdU and PDGFRα in calluses at 2 weeks after the operation. (C) Quantification of PCNA^+^ cells in calluses at 2 weeks after the operation. ****p < 0.0001 versus control, *n* = 6. (D) Quantification of EdU^+^ cells in calluses at 2 weeks after the operation. **p < 0.01 versus control, *n* = 3. (E) Quantification of EdU^+^PDGFRα^+^ cells in calluses at 2 weeks after the operation. ***p < 0.001 versus control, *n* = 3. (F, G) mRNA levels of proliferation‐related genes (*Ki67 and Pcna*) in the cKO mice. *p < 0.05, **p < 0.01 versus control, *n* = 3. (H) EdU assay of C3H10T1/2 cell lines in the *Bmpr1a*‐siRNA group and its statistical analysis. ****p < 0.0001 versus control, *n* = 10. (I) CCK‐8 assay of C3H10T1/2 cell lines after transfection with *Bmpr1a‐siRNA, n* = 6. (J) Colony formation assay of C3H10T1/2 cell lines after transfection with *Bmpr1a*‐siRNA and statistical analysis. ****p < 0.0001 versus control, *n* = 6. (K, L) mRNA levels of proliferation‐related genes (*Ki67 and Pcna*) in the *Bmpr1a*‐siRNA group. **p < 0.01 versus control, *n* = 4.

The number of osteoblasts or preosteoblasts is governed by a balance between proliferation and apoptosis [30, 31, 32]. PCNA increased in *Bmpr1a* cKO calluses, which was mainly around new bone (Figure 4A,C). EdU labeling revealed a higher proportion of proliferating cells in the callus of BMPR1A‐deficient mice compared with controls, further supporting the enhanced proliferative activity during early bone regeneration (Figure 4B,D). Co‐immunostaining for EdU and PDGFRα further showed increased PDGFRα^+^ proliferating cells in the cKO group (Figure 4B,E), indicating expansion of proliferating OPCs. *Pcna*(Figure 4F) and *Ki67* (Figure 4G)were upregulated in *Bmpr1a* cKO mice, whereas TUNEL, BAX/BAK did not differ in 2 groups (Figure S3C). These findings suggested that BMPR1A primarily regulates the proliferation rather than apoptosis of osteolineage cells. We next used C3H10T1/2 cells, a murine OPC line with high BMPR1A expression [33, 34], and established BMPR1A‐knockdown (*Bmpr1a*‐KD) cells by siRNA. EdU assays showed a 1.6‐fold proliferation increase (Figure 4H, *p* < 0.005), CCK‐8 and colony formation assays confirmed enhanced proliferation (Figure 4I,J), and RT‐qPCR showed upregulated *Pcna* and *Ki67* (Figure 4K,L). Conversely, *Bmpr1a* overexpression suppressed proliferation (Figure S4A,B). Importantly, co‐transfection of *Bmpr1a*‐siRNA with the overexpression plasmid rescued altered proliferation to base line (Figure S4C), comfirmed BMPR1A as the regulator of OPC proliferation.

Besides, to investigate whether changes in the ligand microenvironment contribute to the enhanced OPC proliferation in BMPR1A cKO mice, we examined the expression of canonical BMP ligands (*Bmp2*, *Bmp4*) and quiescence‐related factors (*Oct4*, *Nanog*) in callus tissues at 2 weeks. qRT‐PCR analysis revealed no significant differences in any of these genes between control and cKO mice (Figure S4D). These results indicated that the enhanced proliferation is not driven by altered ligand expression, but rather by loss of BMPR1A‐mediated signal interpretation in OPCs, further supporting the cell‐autonomous role of BMPR1A signalling in regulating OPC proliferation.

### 3.5 BMPR1A Regulates OPC Proliferation Mainly Through the SMAD1/5/9 Signalling Cascade and the Transcription Factor *Id1*

**FIGURE 5:**
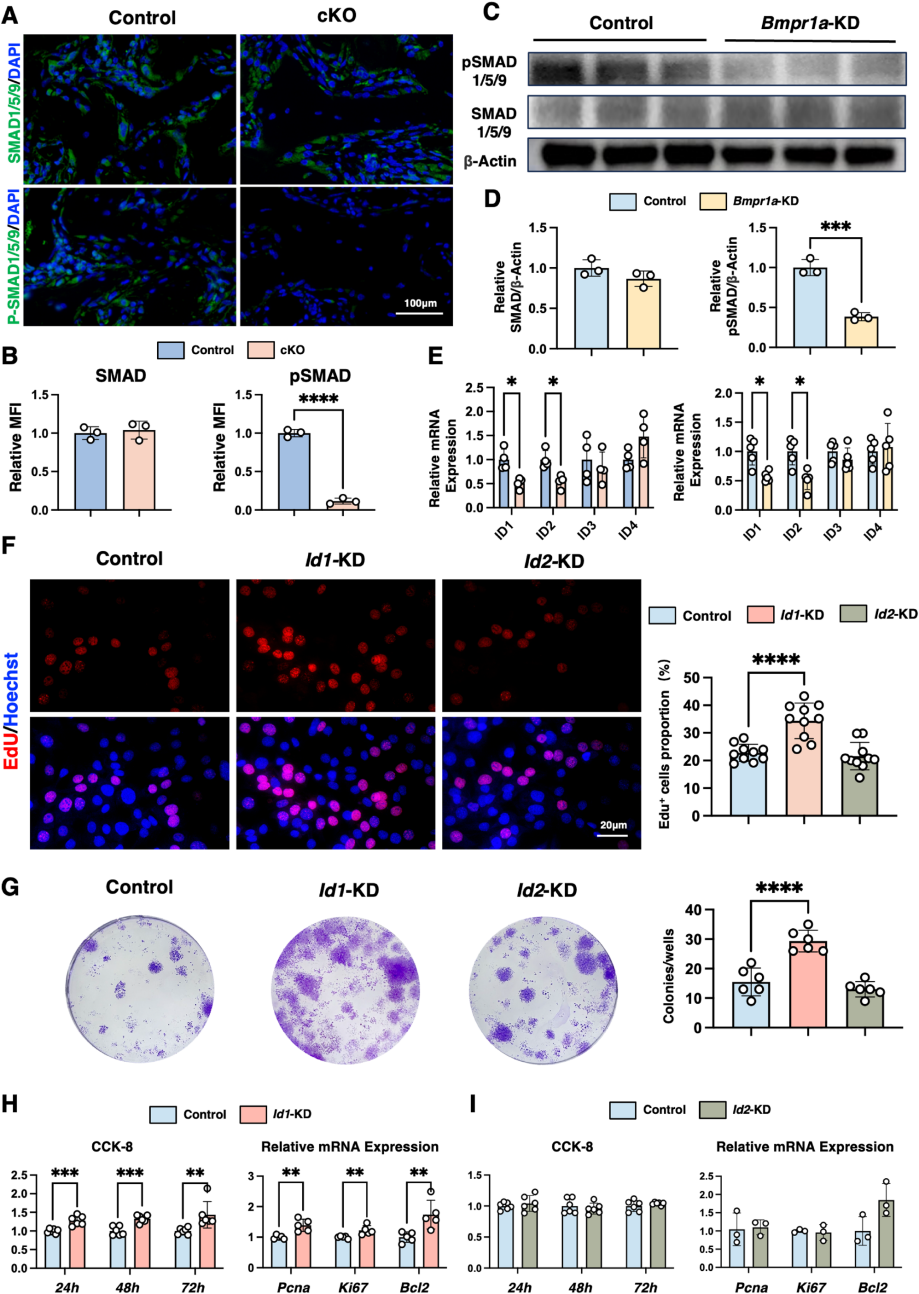
BMPR1A regulated the proliferation of OPCs mainly through the intracellular SMAD1/5/9 signalling cascade and the transcription factor *Id1*. (A, B) Immunofluorescence staining of SMAD1/5/9 and P‐SMAD 1/5/9 in calluses and its statistical analysis. (C, D) Western blot analysis showing the expression of SMAD1/5/9 and pSMAD1/5/9 in *Bmpr1a–KD* cell lines and its statistical analysis, *n* = 3. (E) mRNA levels of *ids* genes (Id1, Id2, Id3, Id4) in cKO mice (*n* = 4) and *Bmpr1a*‐KD cell (*n* = 5). *p < 0.05 versus control. (F) EdU assay of C3H10T1/2 cell lines after transfection with *Id1*‐siRNA or *Id2*‐siRNA and statistical analysis of the results. ****p < 0.0001 versus control, *n* = 10. (G) Colony formation assay of C3H10T1/2 cell lines after transfection with *Id1*‐siRNA or *Id2*‐siRNA and statistical analysis. ****p < 0.0001 versus control, *n* = 6. (H) CCK‐8 assay (*n* = 6) and mRNA (*n* = 5) levels of proliferation‐associated genes after transfection with *Id1*‐siRNA. *p < 0.05 versus control. (I) CCK‐8 assay (*n* = 6) and mRNA (*n* = 3) levels of proliferation‐associated genes after transfection with *Id2*‐siRNA.

The canonical SMAD signalling pathway is a well‐established cascade downstream of BMPR1A [11, 13, 14]. Immunofluorescence staining revealed a marked decrease in pSMAD1/5/9 levels in *Bmpr1a*‐cKO calluses (Figure 5A,B), and Western blotting showed a similar trend in *Bmpr1a*‐KD cells (Figure 5C,D), confirming the suppression of SMAD signalling upon BMPR1A inhibition. Conversely, BMPR1A overexpression elevated pSMAD1/5/9 levels (Figure S5A), further confirming that BMPR1A positively regulates SMAD signalling.

The ID family (including ID1‐4), which act as dominant‐negative antagonists of basic helix–loop–helix (bHLH) transcription factors by competitively inhibiting their binding to E‐box motifs (CANNTG), is a critical downstream target of SMAD signalling [35, 36, 37]. RT–qPCR analysis revealed that *Id1* and *Id2* were significantly downregulated in *Bmpr1a* cKO calluses (Figure 5E), which was consistent with *the* in vitro results (Figure 5E and Figure S5B). To further test their role, we generated *Id1*‐KD and *Id2*‐KD C3H10T1/2 cells. CCK‐8, EdU, and colony formation assays revealed that *Id1*‐KD significantly increased cell proliferation (Figure 5F–I), which was accompanied by increases in *Pcna* (1.38‐fold), *Ki67* (1.22‐fold), and *Bcl2* (1.74‐fold). Western blotting confirmed a 50% reduction in ID1 in *Bmpr1a* KD cells (Figure S5C, *p* < 0.005). Colony formation assays indicated that the cell proliferation capacity was increased in the *Id1*‐KD group, which partially recapitulated the role of *Bmpr1a*, whereas *Id2*‐KD had no significant effect.

Collectively, these findings suggested that ID1, regulated by BMPR1A‐SMAD1/5/9 signalling, is a key mediator of OPC proliferation.

### 3.6 Diminishing the Activity of the BMPR1A‐Mediated Signalling Pathway in OPCs Led to Increased GNG4 Expression and PI3K‐AKT Signalling Activity

**FIGURE 6:**
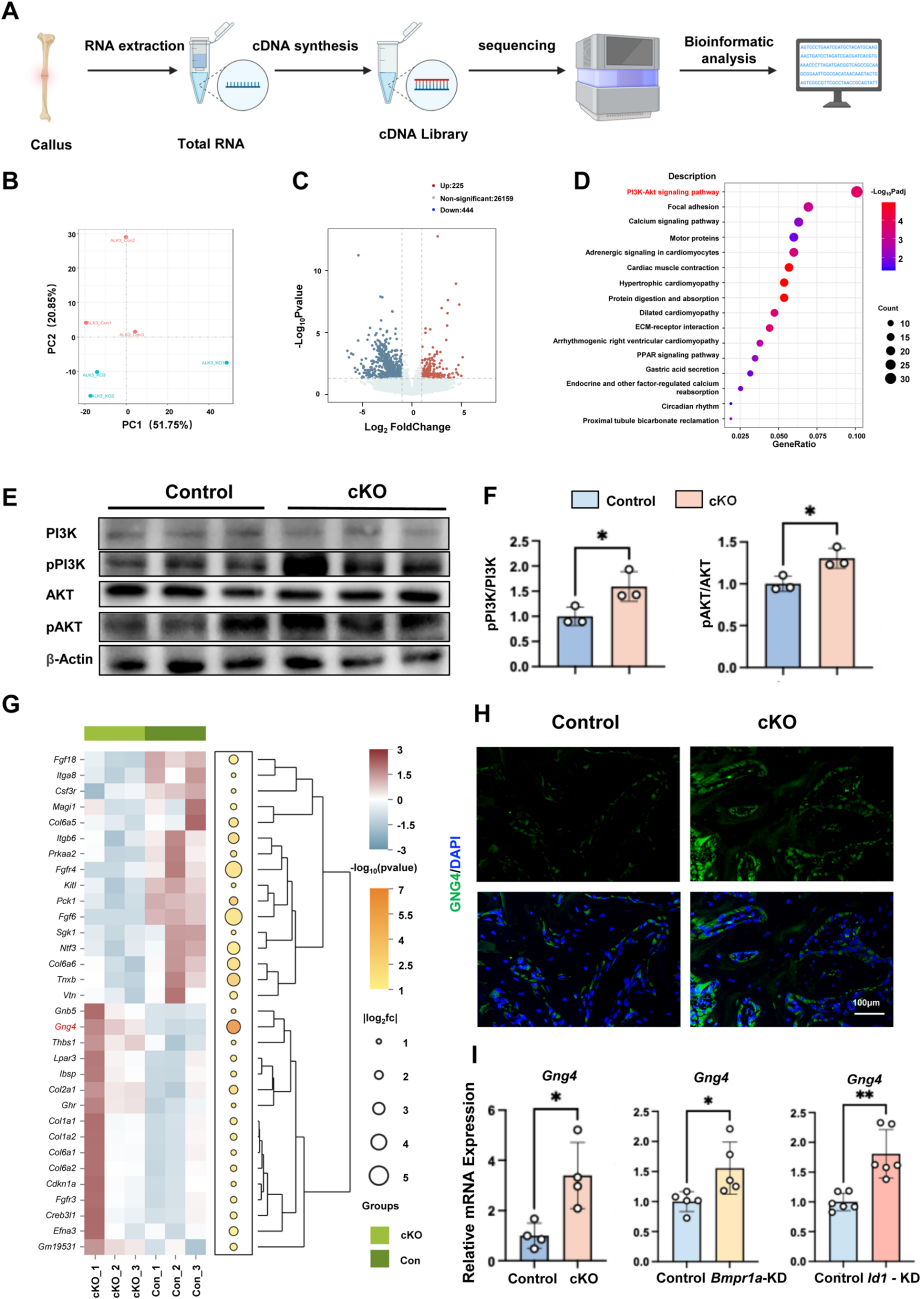
RNA‐Seq revealed significant upregulation of the Pi3K‐AKT pathway and *Gng4* in bone regeneration. (A) The schematic diagram showing the work flow of RNA sequencing. (B) Principal component analysis of the two different groups (*n* = 3 per genotype). (C) Volcano plots showing differentially expressed genes in callus harvested from the control group and cKO group. (D) Kyoto Encyclopedia of Genes and Genomes (KEGG) gene set enrichment analysis revealed several pathways associated with BMPR1A in the process of bone repair. (E—F) Western blot analysis showing the expression levels of PI3K and AKT in callus tissues from control and BMPR1A cKO mice and its statistical analysis, *p < 0.05 versus control, *n* = 3. (G) Differentially expressed genes associated with the PI3K‐AKT signalling pathway according to the RNA‐seq results. (H) Immunofluorescence staining of GNG4 in calluses at PD 2 weeks. (J) mRNA level of *Gng4* in cKO mice (*n* = 4), *Bmpr1a*‐KD (*n* = 5) and *Id1*‐KD (*n* = 6) cell lines. *p < 0.05, **p < 0.01 versus control.

To elucidate the mechanisms underlying the enhanced OPC proliferation in *Bmpr1a*‐cKO mice, *RNA‐seq* of calluses was performed as illustrated in Figure 6A. The principal component analysis (PCA) showed that two groups were clustered separately, demonstrating that these groups exhibited markedly different expression profile characteristics (Figure 6B). Differentially expressed genes (DEGs) were exhibited by volcano map, showing 225 up‐ and 444 downregulated genes compared with those from control mice (Figure 6C). KEGG pathway enrichment highlighted the significant involvement of the PI3K‐AKT signalling, focal adhesion, and calcium signalling pathways, with the PI3K‐AKT signalling pathway being the most significantly altered pathway (*p* < 0.001, Figure 6D). Given the established role of PI3K‐AKT in osteoprogenitor proliferation [38, 39], we further validated the activation of PI3K‐AKT signalling via Western blot analysis. Western blotting confirmed elevated pPI3K and pAKT levels in cKO callus (Figure 6E,F). We subsequently focused on PI3K‐AKT‐associated differentially expressed genes and identified *Gng4* as the most significantly upregulated gene (Figure 6G). As a G protein‐coupled receptor (GPCR) subunit, GNG4 mediates PI3K‐AKT activation [40, 41, 42]. Immunofluorescence staining of the callus revealed enhanced GNG4 expression along the new formed bone surface, overlapping with OPCs (Figure 6H). Consistently, *Gng4* expression was elevated in the callus tissue and further increased in both *Bmpr1a*‐KD and *Id1*‐KD cells (Figure 6I), indicating that BMPR1A–ID1 signalling negatively regulates GNG4 expression. To directly assess the functional role of GNG4 in PI3K‐AKT activation, we knocked down *Gng4* in C3H10T1/2 cells and observed significant reductions in both pPI3K and pAKT, while PI3K and AKT remained unchanged (Figure S5D), confirming that GNG4 is required for activating PI3K‐AKT signalling. Together, these results identified GNG4 as a downstream effector of the BMPR1A–SMAD–ID1 pathway that activates PI3K–AKT signalling to promote OPC proliferation during bone regeneration.

### 3.7 BMPR1A Regulates OPC Proliferation via the ID1‐TCF3‐GNG4 Axis

**FIGURE 7:**
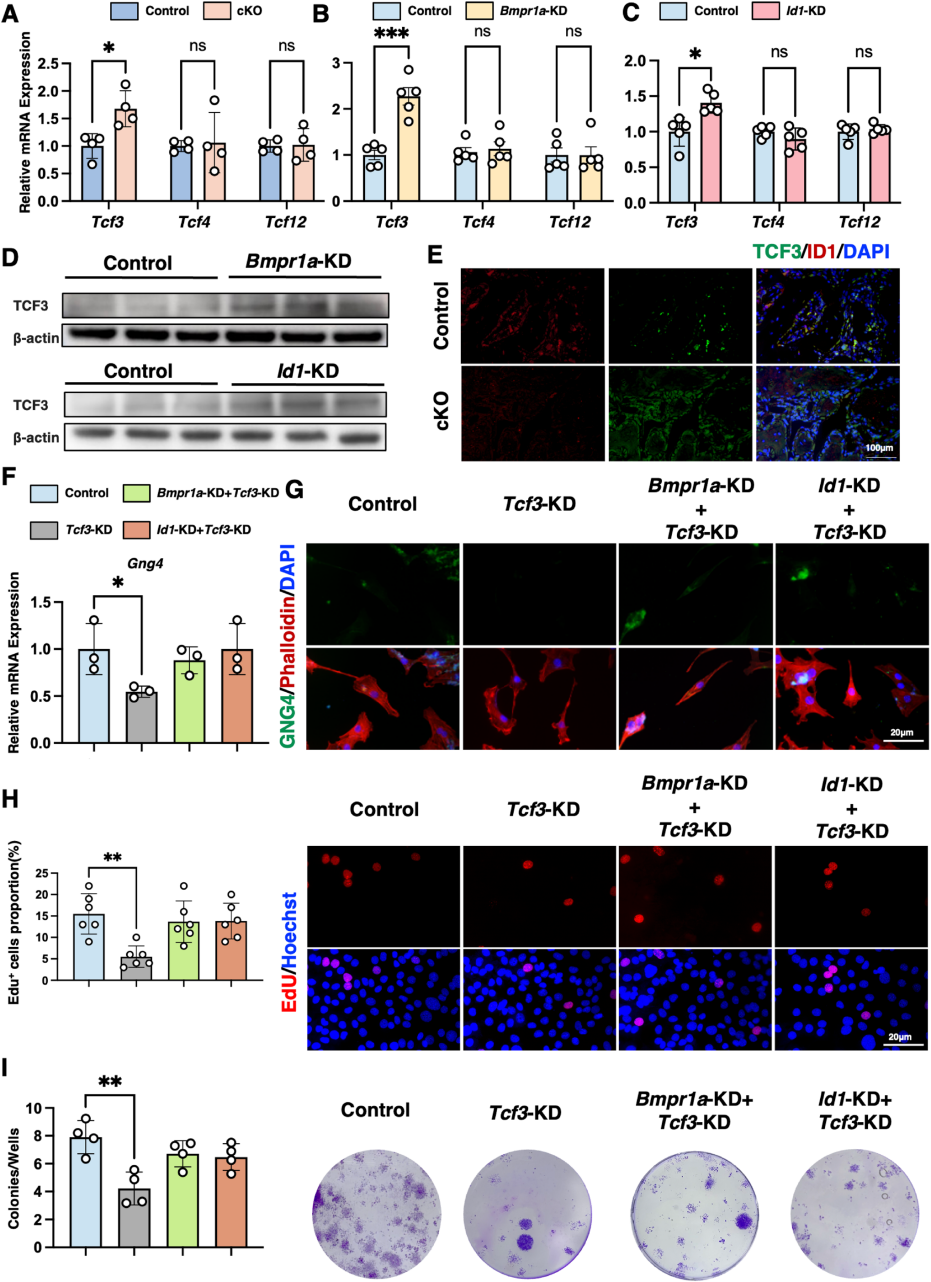
BMPR1A regulates OPC proliferation via the ID1‐TCF‐GNG4 axis. (A–C) mRNA levels of *Tcf* family genes (*Tcf3, Tcf4, and Tcf12*) in vivo (cKO, A, *n* = 4) or in vitro *Bmpr1a* KD (B, *n* = 5) and *Id1* KD (C, *n* = 5). *p < 0.05 versus the control. (D) Western blot results of TCF after transfection with *Bmpr1a*‐siRNA or *Id1*‐siRNA. (E) Immunofluorescence co‐staining of ID1 and TCF3. (F) mRNA expression level of *Gng4* in the different groups. *p < 0.05 versus the control, *n* = 3. (G) Immunofluorescence staining of GNG4 in different groups. (H) EdU assay of the C3H10T1/2 cell line in different groups and statistical analysis of the results. **p < 0.01 versus the control, *n* = 6. (I) Colony formation assay of the C3H10T1/2 cell line in different groups and statistical analysis. **p < 0.01 versus the control, *n* = 4.

Given that ID1 acts as a dominant‐negative antagonist of other transcription factors (TFs), we investigated whether ID1 suppresses *Gng4* via specific TFs. STRING analysis revealed no direct interaction between ID1 and GNG4, but the TCF/LEF (T‐cell factor/lymphoid enhancer factor) family, which is associated with osteogenic activity [36], has the potential to regulate this process (Figure S5E). JASPAR database analysis (MA0521.1 matrix) also predicted potential binding of TCF3/TCF12 to the GNG4 promoter (https://jaspar.elixir.no/). We validated the expression of *Tcf* family members (*Tcf3/4/12*) in *Bmpr1a*‐cKO calluses, *Bmpr1a*‐KD cells, and *Id1*‐KD cells. *Tcf3* was consistently upregulated both in vivo (Figure 7A) and in vitro (Figure 7B,C), whereas *Tcf4* or *Tcf12* showed minimal changes (Figure 7A–C). Western blotting confirmed TCF3 upregulation in *Bmpr1a‐* or *Id1*‐KD cells (Figures 7D and S6). To directly validate this transcriptional regulation, we performed a luciferase reporter assay using a fragment of the *Gng4* promoter. As predicted, TCF3 overexpression significantly enhanced Gng4 promoter activity by ~2‐fold compared with control (Figure S5F).

We next examined the mechanism of TCF3 in vitro. Given the established binding between ID1 and TCF3, we examined their relationship upon BMPR1A loss. *Bmpr1a* inhibition led to reduced ID1–TCF3 colocalization, accompanied by a significant increase in TCF3 (Figure S5G). We also observed that TCF3 was consistently upregulated in cKO calluses. Immunofluorescence staining further confirmed the spatial relationship between these factors, showing altered ID1–TCF3 distribution in cKO mice where TCF3 levels were elevated (Figure 7E), suggesting that TCF3 acts as a downstream of BMPR1A–ID1 to regulate OPC proliferation.

Finally, to confirm the functional necessity of this axis, double knockdown (DKD) of *Tcf3* with *Bmpr1a* KD or *Id1* KD was performed. GNG4 expression was restored to base line (Figure 7F,G), and EdU and colony formation assays showed that cell proliferation returned to normal (Figure 7H,I). Together, these results demonstrated that TCF3 rescues the aberrant proliferation induced by BMPR1A or ID1 inhibition, further highlighting its role as a key downstream transcription factor of ID1.

Collectively, our findings revealed that the BMPR1A‐ID1‐TCF3‐GNG4 signalling axis is linked to PI3K signalling to govern OPC proliferation during bone regeneration, enhancing osteogenesis and accelerating remodelling. Precise modulation of BMPR1A may provide a therapeutic strategy to enhance regeneration.

## 4 Discussion

Our study provides new insights into the role of BMPR1A in regulating OPC and bone regeneration, revealing a novel mechanism of BMP signalling. Conditional knockout of BMPR1A in OPCs during the first 2 weeks accelerated bone regeneration, as evidenced by enhanced cell proliferation, callus formation, and early remodelling. These findings indicate that the first 2 weeks may represent a critical window for BMPR1A‐mediated BMP signalling in regulating bone regeneration, highlighting the importance of temporal precision when targeting this pathway and suggesting that moderate inhibition of BMPR1A‐mediated BMP activity during the early phase may be beneficial for bone repair. In contrast to the traditional view that BMP signalling primarily governs osteogenic differentiation and mineralization, BMPR1A was found to critically regulate OPC proliferation. Notably, we identified an ID1–TCF3–GNG4 signalling axis that links BMP–SMAD signalling to PI3K–AKT activation and revealed how BMP signalling modulates OPC activity during bone repair.

Previous reports have mostly focused on the developmental roles of BMPR1A, yet its function during regeneration remains poorly characterized [43, 44, 45, 46, 47]. Notably, *Bmpr1a* ablation in Osteolineage cells increases trabecular bone mass [43, 46, 47, 48, 49]. paradoxically enhances bone formation despite the expected BMP signalling impairment [11, 13, 14]. This dual effect likely arises, on one hand, from the suppression of BMPR1A‐mediated BMP signalling, which restrains osteogenic activity, and on the other hand, from altered RANKL secretion by osteoprogenitor cells (OPCs), which shifts the RANKL/OPG balance, indirectly modulates osteoclast function, and ultimately impacts bone mass [11, 43, 44, 47, 50]. These findings prompted us to select BMPR1A as a central regulatory mediator, which was subsequently validated in our study.

Unlike these developmental studies, our study reveals a striking increase in OPC proliferation during the first 2 weeks post‐injury, suggesting that an early proliferative burst initiates and accelerates bone regeneration. In addition, BMPR1A knockout in OPCs also led to increased osteoclastic activity. Although the precise mechanism remains unclear, we speculate that the enhanced proliferation drives early bone formation, accelerating the transition into remodelling. Although BMPR1A regulates osteolineage proliferation during development [16, 43, 46, 49], the downstream molecular mechanism remains unclear. *RNA‐seq* and pathway analyses revealed a novel BMPR1A–ID1–TCF3–GNG4 signalling axis that promotes OPC proliferation.

ID proteins are canonical BMP‐SMAD targets acting as dominant‐negative antagonists of bHLH transcription factors [36, 37, 51]. ID1 has dual effects on cell proliferation: it maintains stem cell quiescence and promotes tumour cell proliferation [52, 53], whereas its inhibition stimulates endothelial cell proliferation [54], induces exit from quiescence in zebrafish neural stem cells, and enhances neural stem cell proliferation [55, 56]. These effects likely stem from TFs suppression by ID1. Here, the BMPR1A knockout–induced reduction in *Id1* may relieve this suppression, increase specific TF activity, promote OPC proliferation, and ultimately accelerate bone regeneration.

RNA‐seq and PPI analysis revealed that ID1 downregulation elevates TCF3, a bHLH family member regulating stem cell differentiation and proliferation [36, 57, 58]. We confirmed that ID1 downregulation enhances TCF3 activity, and that TCF3 silencing rescues the enhanced cell proliferation phenotype caused by ID1 deficiency. Notably, RNA‐seq analysis identified *Gng4* as the most significantly differentially expressed gene within the PI3K–AKT signalling pathway.

This study established *Gng4* as a direct functional target of TCF3. We not only observed the correlative expression changes between TCF3 activation and *Gng4* upregulation in the context of *Bmpr1a* knockdown, but also demonstrated, through luciferase reporter assays, that TCF3 transcriptionally activates the *Gng4* promoter. According to previous reports, GNG4, a subunit of the GPCR family, participates in neurotransmission, hormone secretion, cell proliferation, differentiation, and so forth [42]. It is recognized as a cancer detection marker for lung cancer, gastric cancer, colorectal cancer, bladder cancer, and osteosarcoma because of its role in regulating proliferation [59, 60, 61, 62, 63]. An innovative finding of our study is the first identification of GNG4 expression on the surface of OPCs, with enrichment around newly formed bone in cKO mice. Functional experiments further confirmed that *Gng4* knockdown significantly reduced p‐PI3K and p‐AKT, establishing the essential role of GNG4 in the PI3K‐AKT pathway. The high co‐localization of GNG4 with OPCs during bone regeneration suggests that it may accelerate callus formation by promoting OPC proliferation. However, although GNG4 is identified as a novel downstream target of the BMPR1A–ID1–TCF3 signalling axis, its broad expression across various malignancies and relatively lower specificity than BMPR1A indicate that more precise targeting strategies are required.

Interestingly, Julien et al. reported that, in contrast with our bone defect model, BMPR1A inhibits cell proliferation in a fracture model [64], highlighting the influence of model, Cre specificity, and timing of induction on BMPR1A function. These findings underscore the necessity for spatiotemporal precision in BMPR1A during bone regeneration. Several aspects of this study warrant further investigation. Our findings suggest that inhibition of BMPR1A‐mediated BMP signalling during the first 2 weeks of bone regeneration could enhance bone repair. To advance translational potential, we plan to employ sustained‐release materials in animal models to assess the in vivo efficacy of this approach and lay the groundwork for clinical application. What's more, the mechanisms underlying the contrasting effects of BMPR1A on osteoclasts during bone development and bone regeneration remain unclear. It should be noted that the primary goal of this study was not to exhaustively identify all TCF3 targets, but rather to establish the BMPR1A–ID1–TCF3–Gng4 signalling axis. Nevertheless, TCF3 likely orchestrates a broader regulatory network, and systematic identification of additional targets in future studies will provide a more comprehensive understanding of its role in bone regeneration.

Overall, our study reveals a previously unappreciated role of BMPR1A, a key type I BMP receptor and biological macromolecule, in regulating bone regeneration. These findings suggest that temporally controlled, cell‐specific modulation of BMP signalling could provide a novel strategy to enhance bone regeneration while minimizing side effects in clinical BMP therapies.

## Author Contributions

Zihao Zhou designed the research, conducted the experiments, analysed the RNA‐seq data and wrote the manuscript. Yun Zhai conducted the experiments and analysed the data. Xiaochen Liu and Jiaojiao Shao analysed the data and provided suggestions. Jiani Zhou, Zhaoyang Li and Jiale He assisted with the mouse breeding, and Qi Zhang and Shuxian Lin supervised the research and edited the manuscript. All the authors read and approved the final paper.

## Funding

This work was supported by National Natural Science Foundation of China, 82370950, 82170945, 82401093.

## Conflicts of Interest

The authors declare no conflicts of interest.

## Supporting information


**Figure S1:** (A)Gating strategy recommended to sort the different cells of stem cell lineage; (B) Immunofluorescence staining shows co‐localization (yellow) of PDGFRα (green) and tdTomato (red) in the bone of control mice. (C) Immunofluorescence staining of BMPR1A in three groups of mice.
**Figure S2:** (A‐B) MicroCT of cKO and CA TM0‐1 weeks, 0–3weeks, 0–4 weeks femurs which harvested at 4 weeks after operation compared with control group (Scale bar = 200 μm) and statistical analysis of BV/TV, *n* = 3; (C) HE staining in three groups of mice at 4 weeks post‐operation.; (D) Statistical analysis of Tb.Th and BMD from MicroCT of the callus of 3 groups at 2 weeks after operation, *n* = 6.
**Figure S3:** (A) Immunofluorescence staining of SP7 and OCN in control mice. (Upper) SP7 (green) staining in the callus at 1 week post‐operation. (Lower) OCN (green) staining in the callus at 4 weeks post‐operation. (B) Trap staining and immunofluorescence staining of RANKL and OPG in control and cKO mice at 2 weeks post‐operation; (C) TUNEL staining and immunofluorescence staining of BAX and BAK in control and cKO mice at 2 weeks post‐operation.
**Figure S4:** (A) EDU staining (red) in control and OE cells. OE group showed fewer EDU‐positive cells. *p < 0.05, *n* = 7. (B) Proliferation genes (Ki67, Pcna) were downregulated in OE cells. *p < 0.05, *n* = 3. (C) No difference in proliferation genes after co‐transfection with BMPR1A‐OE and siRNA, *n* = 3. (D) No difference in BMP ligands and quiescence‐related genes in cKO mice versus controls, *n* = 3.
**Figure S5:** (A) Western blot analysis of pSMAD protein levels in control and OE cells with quantification. **p < 0.01, *n* = 3. (B) qPCR analysis of ID1‐4 mRNA expression in OE cells compared with controls. *p < 0.05, *n* = 3. (C) Western blot analysis of ID1 protein levels after BMPR1A‐siRNA treatment. **p < 0.01, *n* = 3. (D) Western blot analysis of PI3K‐AKT pathway changes upon *Gng4* knockdown. (E) Predicted protein–protein interaction network for ID1 from STRING database. (F) Luciferase reporter assay showing TCF3 binding to GNG4 promoter. (G) Reduced binding between ID1 and TCF3 after BMPR1A knockdown.
**Figure S6:** Statistical analysis of western blotting showed the expression levels of TCF3 in different groups. *p < 0.05 versus relative control, *n* = 3.
**Table S1:** List of primers used and the respective forward and reverse sequences.
**Table S2:** List of forward and reverse sequences of siRNA.

## Data Availability

Some or all data that support the findings of this study are available from the corresponding author upon reasonable request.
