# Supplementary material for: Unexpected Promotion of Bone Regeneration by Inhibition of BMPR1A‐Mediated BMP Signalling
Source: Cell Prolif. 2026 Apr 6;59(8):e70204. doi: 10.1111/cpr.70204 (PMC13325999; doi:10.1111/cpr.70204)
Supplement: Supplementary file 1 [file CPR-59-e70204-s001.docx]

**Supplementary Figures**

**Supplementary Figure 1** **(A)**Gating strategy recommended to sort the different cells of stem cell lineage; **(B)** Immunofluorescence staining shows co-localization (yellow) of PDGFRα (green) and tdTomato (red) in the bone of control mice. **(C)** Immunofluorescence staining of BMPR1A in three groups of mice;

**Supplementary Figure 2 (A-B)** MicroCT of cKO and CA TM0-1wks,0-3wks, 0-4wks femurs which harvested at 4wks after operation compared with control group (Scale bar=200μm) and and statistical analysis of BV/TV, n=3; **(C)** HE staining in three groups of mice at 4 weeks post-operation.;**(D)** Statistical analysis of Tb.Th and BMD from MicroCT of the callus of 3 groups at 2wks after operation, n=6.

**Supplementary Figure 3（A）**Immunofluorescence staining of SP7 and OCN in control mice. (upper) SP7 (green) staining in the callus at 1 week post-operation. (lower) OCN (green) staining in the callus at 4 weeks post-operation.**（B）**Trap staining and immunofluorescence staining of RANKL and OPG in control and cKO mice at 2 weeks post-operation; **(C)** TUNEL staining and immunofluorescence staining of BAX and BAK in control and cKO mice at 2 weeks post-operation

**Supplementary Figure****4 (A)** EDU staining (red) in control and OE cells. OE group showed fewer EDU-positive cells. *P < 0.05, n=7. **(B)** Proliferation genes (Ki67, Pcna) were downregulated in OE cells. *P < 0.05, n=3.**(C)** No difference in proliferation genes after co-transfection with BMPR1A-OE and siRNA, n=3. **(D)** No difference in BMP ligands and quiescence-related genes in cKO mice versus controls, n=3.

**Supplementary Figure 5**  (A) Western blot analysis of pSMAD protein levels in control and OE cells with quantification. **P < 0.01,n=3. (B) qPCR analysis of ID1-4 mRNA expression in OE cells compared to controls. *P < 0.05, n=3. (C) Western blot analysis of ID1 protein levels after BMPR1A-siRNA treatment. **P < 0.01, n=3. (D) Western blot analysis of PI3K-AKT pathway changes upon *Gng4* knockdown. (E) Predicted protein-protein interaction network for ID1 from STRING database. (F) Luciferase reporter assay showing TCF3 binding to GNG4 promoter. (G) Reduced binding between ID1 and TCF3 after BMPR1A knockdown.

**Supplementary Figure 6.** Statistical analysis of western blotting showed the expression levels of TCF3 in different groups. **p* < 0.05 versus relative control, n=3.

**Tables**

Supplementary Table 1. List of primers used and the respective forward and reverse sequences.

| Gene | Forward sequences | Reverse sequences |
| --- | --- | --- |
| *Bmpr1a* | 5'- TGGCACTGGTATGAAATCAGAC -3' | 5'- CAAGGTATCCTCTGGTGCTAAAG -3' |
| *Ki67* | 5'- ATCATTGACCGCTCCTTTAGGT -3' | 5'- GCTCGCCTTGATGGTTCCT -3' |
| *Pcna* | 5'- TTTGAGGCACGCCTGATCC -3' | 5'- GGAGACGTGAGACGAGTCCAT -3' |
| *Bcl2* | 5'- GTCGCTACCGTCGTGACTTC -3' | 5'- CAGACATGCACCTACCCAGC -3' |
| *Id1* | 5'- CCTAGCTGTTCGCTGAAGGC -3' | 5'- CTCCGACAGACCAAGTACCAC -3' |
| *Id2* | 5'- ATGAAAGCCTTCAGTCCGGTG -3' | 5'- AGCAGACTCATCGGGTCGT -3' |
| *Id3* | 5'- CTGTCGGAACGTAGCCTGG -3' | 5'- GTGGTTCATGTCGTCCAAGAG -3' |
| *Id4* | 5'- CAGTGCGATATGAACGACTGC -3' | 5'- GACTTTCTTGTTGGGCGGGAT -3' |
| *Gng4* | 5'- GGCATGTCTAATAACAGCACCA -3' | 5'- CACTGGGATGATGAGGGGG -3' |
| *Tcf3* | 5'- GGGTGCCAGCGAGATCAAG -3' | 5'- ATGAGCAGTTTGGTCTGCGG -3' |
| *Tcf4* | 5'- CGAAAAGTTCCTCCGGGTTTG -3' | 5'- CGTAGCCGGGCTGATTCAT -3' |
| *Tcf12* | 5'- ATGTACTGTGCTTATCCTGTCCC -3' | 5'- GGTGCATATACCGTTTTCCCATT -3' |
| *β-actin* | 5'- GGCTGTATTCCCCTCCATCG -3' | 5'- CCAGTTGGTAACAATGCCATGT -3' |

Supplementary Table 2. List of forward and reverse sequences of siRNA.

| Gene | Forward sequences | Reverse sequences |
| --- | --- | --- |
| *Bmpr1a* | 5'- CGAUGAAUGUCUUCGAGCA(dT)(dT) -3' | 5'- UGCUCGAAGACAUUCAUCG(dT)(dT) -3' |
| *Id1* | 5'- GCGAGGUGGUACUUGGUCUGU(dT)(dT) -3' | 5'-ACAGACCAAGUACCACCUCGC(dT)(dT) -3' |
| *Id2* | 5'- GAGCUUAUGUCGAAUGAUA(dT)(dT) -3' | 5'- UAUCAUUCGACAUAAGCUC(dT)(dT) -3' |
| *Tcf3* | 5'- CCGGAUCACUCCAGCAAUA(dT)(dT) -3' | 5'- UAUUGCUGGAGUGAUCCGG (dT)(dT) -3' |
| *Gng4* | 5'- AGGAAGGCAUGUCUAAUAA(dT)(dT) -3' | 5'- UUAUUAGACAUGCCUUCCU(dT)(dT) -3' |
